# Rates and Causes of Readmission Within 60 Days Following Hysterectomy in a Tertiary Care Center in Saudi Arabia

**DOI:** 10.7759/cureus.36500

**Published:** 2023-03-22

**Authors:** Nashwa AlDardeir, Ghadi Alzhrani, Abdulsalam Alqutub, Raghad Kabli, Deyala Sait, Refan Alsaeed, Shahad Alruwaithi, Areej Algarni, Dana Sawan

**Affiliations:** 1 Department of Obstetrics and Gynecology, King Abdulaziz University Faculty of Medicine, Jeddah, SAU; 2 Department of Medicine, King Abdulaziz University Faculty of Medicine, Jeddah, SAU

**Keywords:** hysterectomy, causes, predictors, risk factors, retrospective record review, unplanned readmission, hospital readmission rate

## Abstract

Introduction: Unplanned readmissions are frequent, costly, and perhaps avoidable. We aim to identify the rate, causes, and predictive factors of hospital readmission after hysterectomy within 60 days post-discharge at King Abdulaziz University Hospital (KAUH).

Methods: Retrospective record review of all patients who underwent hysterectomy for benign and malignant conditions from January 2017 to December 2022. Patients were evaluated for demographics, comorbidities, and causes for readmission.

Results: Of 117 patients, the unplanned readmission rate was 9.4% and 7.7% for benign and malignant conditions, respectively. Infections (25%) and abdominal pain (20%) were common causes. Patients with increased intraoperative bleeding (P = 0.013) and cancer patients (0.044) had an increased risk for readmission. Readmitted patients had significantly higher baseline health burdens when compared to non-readmitted patients using the American Society of Anesthesiology scores (ASA) (p = 0.011) and the Cumulative Illness Rating Scale (CIRS) (p = 0.004).

Conclusion: The 60-day readmission rate after a hysterectomy was 17.1%. Infections and abdominal pain represented common causes. Malignancy and increased intraoperative blood loss are significant risk factors. In order to decrease the readmission rate, addressing common reasons may be beneficial.

## Introduction

Unplanned hospital visits remain a significant contributor to healthcare expenditure [1]. According to data in the literature, one in every seven patients who underwent a major surgery will be readmitted within 30 days after hospital discharge [2]. The Medicare Payment Advisory Committee states that 12.3% of readmissions are avoidable [3]. Moreover, unplanned readmissions may furtherly expose patients to hospital-acquired complications [4]. Thus, unplanned readmission rates are regarded as a scale of hospitals' quality of care [1,2]. Only second to cesarean section, hysterectomy is the most common surgical procedure worldwide [5]. Nearly one in three American women will have had a hysterectomy by age 60 [6,7].

Previous studies have investigated the rate, predictors, and causes of hospital readmission following hysterectomy, with rates ranging from 3.1% to 13.2% [5,8-10]. One retrospective study concluded that infections were responsible for 42.6% of readmissions [8]. Another study identified gastrointestinal symptoms (38%) and infectious etiologies (34%) as common causes for readmission after a hysterectomy, with predictors for readmission being increased intraoperative blood loss and malignancy as an indication for the hysterectomy [5].

Nevertheless, the rate and causes of hospital readmission after hysterectomy are yet to be described clearly in the Saudi population, as similar data are lacking in our locality. Given the significant impact of hospital readmissions on patients and the healthcare system, identifying modifiable risk factors and preventable causes of unplanned hospital visits may aid in lowering readmission rates. We aim to study the rate and causes of unplanned hospital visits within 60 days of hospital discharge following a hysterectomy at King Abdulaziz University Hospital (KAUH).

## Materials and methods

After obtaining ethical approval from our hospital's Institutional Review Board (IRB) (reference number: 507-22), we retrospectively identified 117 patients who underwent hysterectomies from January 2017 to December 2022. All patients with missing data were excluded, such as those having no documented cause for hospital readmission. All patients were at least 15 years of age.

Our primary outcome was identifying the rate and causes of post-hysterectomy readmission within 60 days. If more than one readmission episode was identified, only the first episode was included in the study. Secondarily we sought to describe the predictors for hospital readmission.

We obtained the medical record number, age, body mass index (BMI), marital status, and reproductive history. Moreover, indication for surgery, surgical approach (vaginal, abdominal, or laparoscopic hysterectomy), condition type, whether benign or malignant, the amount of blood loss in mL, and the dates of primary admission, discharge, and readmission were also collected. Additionally, comorbidities were identified and scored using the American Society of Anesthesiology (ASA) and the Cumulative Illness Rating Scale (CIRS) comorbidity scores.

Google Forms (Google, Inc., Mountain View, CA, USA) was used for data entry. Data was then extracted into Excel version 16.0 (Microsoft Corp., Redmond, WA, USA). Analysis was performed using the Statistical Package for the Social Sciences for Windows version 21.0 (IBM SPSS Statistics, Armonk, NY, USA). In describing continuous variables, means and standard deviation were used for parametric data, and medians and interquartile range (IQR) for non-parametric data. Numbers and percentages were used for categorical variables. Student t-test, Chi-square, and Mann-Whitney U tests were used for bivariate analysis. For all tests, statistical significance was set at P < 0.05.

## Results

Overall, 117 patients underwent a hysterectomy in KAUH between 2017 and 2022; 20 (17.1%) were readmitted within 60 days following discharge, of which 11 (9.4%) were benign cases. Most cases (65%) were readmitted within the first two weeks of discharge. Table 1 summarizes the baseline characteristics and patients' demographic data.

**Table 1:** Patients’ baseline characteristics, demographic data, and surgical approach. CIRS: Cumulative Illness Rating Scale ASA: American Society of Anesthesiology BMI: body mass index

Variable	Included (n = 117)
Age in years (mean ± SD)	60.66 ± 12.48
Length of primary stay in days (median, IQR)	5, (4-7)
CIRS (mean ± SD)	3.3 ± 2.58
ASA score (mean ± SD)	2.06 ± 0.62
Type of condition (n, %)	
Benign	11 (9.4%)
Malignant	9 (7.7)
BMI (n, %)	
<18.5	6 (5.1)
18.5-24.9	11 (9.4)
25-29.9	25 (21.4)
>30	75 (64.1)
Reproductive history (n, %)	
Primigravida	34 (29.1)
Multigravida	69 (59)
Nulliparous	14 (12)
Surgical approach (n, %)	
Laparoscopic	6 (5.1)
Vaginal	67 (57.3)
Abdominal (total hysterectomy)	40 (34.2)
Abdominal (subtotal hysterectomy)	4 (3.4)

Table 2 summarizes the causes for readmission in malignant cases; the single most common cause for readmission was infection (44.44%), including surgical site infection (SSI), urinary tract infection (UTI), and sepsis. Causes for readmission in benign cases are listed in Table 3, with pain being the most frequent cause (36.4%).

**Table 2:** Rates and causes of readmission for malignant cases. SSI: surgical site infection UTI: urinary tract infection

Causes	Numbers	Rates (%)
Infections: SSI, UTI, sepsis	4	44.44
Gastrointestinal: nausea, vomiting	3	33.33
Fatigue	1	1.11
Wound dehiscence	1	1.11

**Table 3:** Rates and causes of readmission for benign cases. SSI: surgical site infection UTI: urinary tract infection

Causes	Numbers	Rates (%)
Abdominal pain	4	36.4
Hematoma	3	27.3
Vaginal discharge	3	18.2
Infections: SSI, UTI, sepsis	1	1.11

Table 4 compares readmitted patients with non-readmitted patients. Women with malignancies were over twice as likely to be readmitted (45% vs. 20.6%; P = 0.044). Moreover, those who lost more blood during the surgery were more likely to return to have an unplanned readmission post-surgery (P = 0.013). Additionally, those who were readmitted had significantly higher ASA scores (2.5 ± 0.83) than those who were not (1.97 ± 0.53), P = 0.011. Similarly, the CIRS scores were significantly higher in readmitted patients (5.1 ± 2.79) than in non-readmitted patients (2.93 ± 2.39), P = 0.004. A statistically significant positive correlation was noted when using simple linear regression between the two measures, P <0.001, with r2 = 0.299.

**Table 4:** Comparison between readmitted and non-readmitted patients in age, BMI, type of condition, previous history of cesarean section, surgical approach, blood loss in mL, ASA score, and CIRS score. BMI: body mass index ASA: American Society of Anesthesiology CIRS: Cumulative Illness Rating Scale

Variable	Readmitted	Non-readmitted	P value
Age			
Mean (SD)	60.93 (12.27)	59.35.20 (13.69)	0.637
BMI (n, %)			
<18.5	1 (5)	5 (5.2)	0.705
18.5-24.9	1 (5)	10 (10.3)	
25-29.9	3 (15)	22 (22.7)	
>30	15 (75)	60 (61.9)	
Type of condition (n, %)			
Benign	11 (55)	77 (79.4)	0.044
Malignant	9 (45)	20 (20.6)	
History of cesarean section (n, %)			
Yes	13 (65)	43 (44.3)	0.15
No	7 (35)	54 (55.7)	
Surgical approach (n, %)			
Laparoscopic	1 (5)	5 (5.2)	0.781
Vaginal	11 (55)	56 (57.7)	
Abdominal (total hysterectomy)	8 (40)	32 (33)	
Abdominal (subtotal hysterectomy)	0 (0)	4 (4.1)	
Blood loss in mL			
Mean (SD)	712.5 (480.92)	412.89 (286.8)	0.013
ASA			
Mean (SD)	2.5 (0.83)	1.97 (0.53)	0.011
CIRS			
Mean (SD)	5.1 (2.79)	2.93 (2.39)	0.004

## Discussion

Although the readmission rate is considered a metric scale for quality of care, non-modifiable risk factors such as age, gender, race, and socioeconomic status may be potential limitations. These factors can contribute to unplanned hospital return and readmission [11].

Our study showed a rate of unplanned hospital readmission of 17.1%. This rate is comparable to rates reported in other studies [8,10]. Jennings et al. studied the rate and predictive factors associated with readmission after laparoscopic hysterectomy, with a rate of 3.1% [9]. This rate is lower than the rate reported in our study. This finding may be because our study incorporated abdominal and vaginal route hysterectomy in addition to laparoscopy. Although our study did not show significant differences in the readmission rate between different surgical routes.

We identified infections, including SSI, UTI and sepsis, and abdominal pain as common causes for readmission. Similar causes have been described, with infections being a frequent cause [9]. To us, these represented preventable causes, as patient and caregiver education and proper discharge planning are important factors to target. Regarding predictive factors, our study revealed an increased risk for readmission following a hysterectomy in patients with more intraoperative blood loss and those with malignancy as an indication for hysterectomy. Similarly, Lee et al. noted increased intraoperative blood loss and cancer as an indication for surgery as significant risk factors for readmission [5]. For patients with such risk factors, earlier proactive outpatient management and screening for the previously mentioned causes of readmission during follow-ups may lower the unplanned readmission rates.

ASA score is confirmed to be closely linked to expecting unplanned readmissions and is positively associated with an increased readmission rate [12-14]. Additionally, the CIRS score in patients undergoing surgery has been used to reflect baseline health deterioration [15]. This signified that readmitted patients had an increased baseline health burden, predisposing them to more severe complications and frequent readmissions. Thus, our study reaffirms that ASA and CIRS comorbidity scores are reliable at predicting post-surgical readmission. We advise that patients with higher baseline health burdens should be followed more frequently, as this intervention may lower the readmission rate in these vulnerable populations. However, these comorbidity scores have not yet been validated to predict readmission after a hysterectomy. We urge researchers to investigate both scores' validity in predicting readmission after a hysterectomy.

Our study is unique in extending the analysis period to 60 days after surgery rather than the usual 30 days, thereby providing exclusive data on postoperative complications and utilization of healthcare resources. Furthermore, our study was conducted in a tertiary academic healthcare center, and many cases are referred to our hospital from peripheral areas; such a process may delay follow-ups. Given our circumstances, extending the analysis period to 60 days post-surgery may give us a better understanding of the actual readmission rate after surgery. Several limitations were faced during the conduction of this study. The usual challenge in retrospective studies to obtain accurate patient data and outcomes was considered a significant limitation. Because of poor documentation, the study did not include critical variables, such as cancer stages in malignant cases and exact operative times. Moreover, the fact that our study was done in a single tertiary care center may raise questions about the generalizability of our data. Thus, future prospective studies on broader scales regarding sample size and demographic data are desired.

## Conclusions

The 60-day readmission rate after a hysterectomy was 17.1%. Malignancy and increased intraoperative blood loss are significant risk factors. Infections and abdominal pain represented common causes. Identifying common causes and risk factors is beneficial. Such data may be used to identify individuals at high risk of readmission after a hysterectomy and to design and execute rate-lowering and quality-improving focused interventions.
